# Diversity and Phylogeny of Cattle Ixodid Ticks and Associated Spotted Fever Group *Rickettsia* spp. in Tunisia

**DOI:** 10.3390/pathogens12040552

**Published:** 2023-04-03

**Authors:** Myriam Kratou, Hanene Belkahia, Rachid Selmi, Rihab Andolsi, Mokhtar Dhibi, Moez Mhadhbi, Lilia Messadi, Mourad Ben Said

**Affiliations:** 1 Laboratory of Microbiology, National School of Veterinary Medicine of Sidi Thabet, University of Manouba, Manouba 2010, Tunisia; 2 Ministry of National Defense, General Directorate of Military Health, Veterinary Service, Tunis 1008, Tunisia; 3 Laboratory of Parasitology, National School of Veterinary Medicine of Sidi Thabet, University of Manouba, Manouba 2010, Tunisia; 4 Department of Basic Sciences, Higher Institute of Biotechnology of Sidi Thabet, University of Manouba, Manouba 2010, Tunisia

**Keywords:** *Rickettsia* species, cattle ticks, molecular species identification, genotyping, phylogenetic analysis, Tunisia

## Abstract

Tick-borne rickettsioses are mainly caused by obligate intracellular bacteria belonging to the spotted fever group (SFG) of the *Rickettsia* genus. So far, the causative agents of SFG rickettsioses have not been detected in cattle ticks from Tunisia. Therefore, the aim of this study was to investigate the diversity and phylogeny of ticks associated with cattle from northern Tunisia and their associated *Rickettsia* species. Adult ticks (*n* = 338) were collected from cattle in northern Tunisia. The obtained ticks were identified as *Hyalomma excavatum* (*n* = 129), *Rhipicephalus sanguineus* sensu lato (*n* = 111), *Hyalomma marginatum* (*n* = 84), *Hyalomma scupense* (*n* = 12) and *Hyalomma rufipes* (*n* = 2). After DNA extraction from the ticks, 83 PCR products based on the mitochondrial 16S rRNA gene were sequenced and a total of four genotypes for *Rh. sanguineus* s.l., two for *Hy. marginatum* and *Hy. excavatum* and only one for *Hy. scupense* and *Hy. rufipes* were recorded, with the occurrence of one, two and three novel genotypes, respectively, for *Hy. marginatum*, *Hy. excavatum* and *Rh. sanguineus* s.l. mitochondrial 16S rRNA partial sequences. The tick DNA was tested for the presence of *Rickettsia* spp. by using PCR measurements and sequencing targeting three different genes (*ompB*, *ompA* and *gltA*). Of the 338 analyzed ticks, 90 (26.6%), including 38 (34.2%) *Rh. sanguineus* s.l., 26 (20.1%) *Hy. excavatum*, 25 (29.8%) *Hy. marginatum* and one (50%) *Hy. rufipes* tick, were positive for *Rickettsia* spp. Based on 104 partial sequences of the three analyzed genes, the BLAST analysis and phylogenetic study showed the infection of *Hy. excavatum*, *Hy. marginatum* and *Rh. sanguineus* s.l. tick specimens with *R. massiliae*, *R. aeschlimannii* and *R. sibirica* subsp. *mongolitimonae* and one *Hy. rufipes* tick specimen with *R. aeschlimannii*. In addition, coinfection with *R. massiliae* and *R. aeschlimannii* was reported in one *Hy. marginatum* and one *Rh. sanguineus* s.l. tick specimen, while a coinfection with *R. massiliae* and *R. sibirica* subsp. *mongolitimonae* was recorded in one *Rh. sanguineus* s.l. tick specimen. In conclusion, our study reports, for the first time in Tunisia, the infection of cattle ticks belonging to *Hyalomma* and *Rhipicephalus* genera with zoonotic *Rickettsia* species belonging to the SFG group.

## 1. Introduction

Ticks are obligate blood-feeding arthropods. They transfer pathogenic bacteria, protozoa and viruses to their vertebrate hosts, such as humans and wild and domestic animals [1]. Different categories of tick-borne diseases, namely babesiosis, ehrlichiosis, anaplasmosis, Lyme disease, Rocky Mountain spotted fever and Crimean Congo hemorrhagic fever, pose a serious threat to animal and human health [1]. Several tick species tolerate and reproduce better in hot and humid climatic conditions [2], in particular the genus *Rhipicephalus*, which has been introduced in several geographical areas around the world, being assisted by its strong capacity to adapt and spread as a vector and its economic impact given its wide distribution, vector capacity, blood-sucking habits and the proportion of cattle that it affects [3,4]. In fact, globally, these ticks affect 80% of the world’s cattle population and are associated with staggering economic losses [5].

The genus *Rickettsia* (family *Rickettsia*ceae; order *Rickettsia*les) is formed by four groups: the typhus group, the spotted fever group (SFG), the *Rickettsia bellii* group and the *Rickettsia canadensis* group [6], causing rickettsioses in vertebrate hosts, including humans and domestic and wild animals [7,8]. These particular vector-borne diseases are transmitted by lice and fleas and constitute a major problem of exceptional importance due to the high morbidity and low mortality rates in humans and animals, as well as their impact on animal production [8]. This situation is enraged by climate change. These changes influence the vectors that transmit pathogens and create conditions that encourage the emergence and re-emergence of numerous diseases, including those spread by ticks. Currently, cattle are the subject of several investigations, since it has been demonstrated that they can act as hosts or reservoirs, like other ruminants, for emerging and re-emerging bacterial infections, including those due to the genera of *Anaplasma* [9,10], *Borrelia* [11], *Bartonella* [12,13], *Coxiella* [14] and *Rickettsia* [15].

The molecular typing of these infectious agents is crucial to better understand ecological niches and identify circulating strains [16]. Therefore, the sequence analysis of PCR-amplified fragments targeting genes encoding *Rickettsia*-specific outer membrane proteins (*ompB*, *ompA*), the citrate synthase (*gltA*) and the ribosomal 16S rRNA gene has become one of the most reliable approaches for the identification of *Rickettsia* species [16,17]. In Tunisia, cases of infections by several *Rickettsia* species have already been reported, such as *R. conorii*, which was the first case of Mediterranean spotted fever (MSF) in humans in 1910 [18], and more recently mentioned by Znazen et al. [18] and Khrouf et al. [19]. Furthermore, several other SFG pathogenic rickettsiae, including *R. helvetica*, *R. africae* and *R. aeschlimannii,* have been revealed in camels and their associated ticks of the *Hyalomma* genus located in the south and center of Tunisia [13,20]. Additionally, *R. massiliae* DNA has been detected in *Rhipicephalus sanguineus* sensu lato ticks collected from dogs [21] and camels [13], and recently from small ruminants reared in the north of the country [22]. So far, the causative agents of SFG rickettsioses have not been detected in cattle ticks in Tunisia. Therefore, such studies would be essential in order to contribute to the knowledge of the current epidemiological situation of rickettsiosis in the country. We investigated the diversity and phylogeny of cattle ticks and associated *Rickettsia* species. As a matter of fact, the *Rickettsia* infection prevalence was evaluated overall according to potential risk factors. Moreover, genotyping and a phylogenetic analysis of the revealed ticks and *Rickettsia* spp. isolates were also carried out using different discriminative gene fragments.

## 2. Materials and Methods

### 2.1. Study Regions, Tick Collection and Morphological Identification

**Figure 1:**
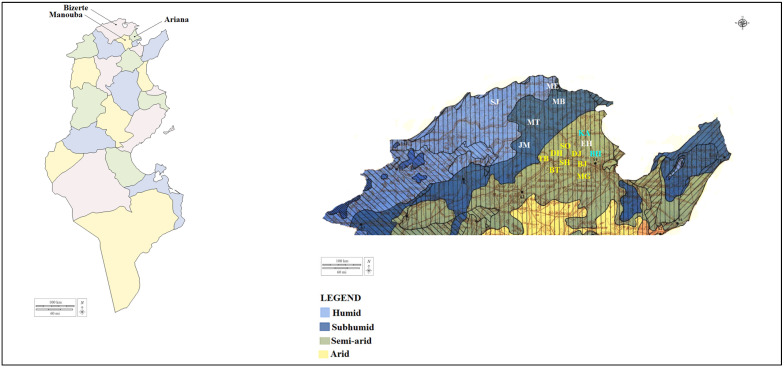
Map of Tunisia showing investigated governorates. Legend: The districts of the governorate of Bizerte are written in white, those of the governorate of Manouba in yellow and those of the governorate of Ariana in blue. Abbreviations: SJ: Sejnane; ME: Metline; MB: Menzel Bourguiba; MT: Mateur; JM: Joumine; EH: El Mabtouh; KA: Kalâat El Andalous; BH: Bach Hamba; SO: Sidi Othmen; DH: Dhniba; TB: Tebourba; BT: El Battan; MG: Mornaguia; BJ: Bjaoua; SH: Sanhaja; DJ: Djedeida.

Between June and September 2019 and 2020, ticks were randomly collected from 254 apparently healthy cattle (189 females and 65 males) reared in 45 farms located in three Tunisian governorates (Bizerte, Ariana and Manouba) belonging to two bioclimatic zones (subhumid and higher semi-arid) (Figure 1). The minimal required number of tick samples was estimated according to the following formula: N = 1.96^2^ ∗ Pexp (1-Pexp)/d^2^ [23]. The expected prevalence (Pexp) of infection was determined according to previous reports on tick-borne bacterial infections in ticks infesting African ruminants (Pexp = 30%) with a confidential interval of 95% [21,23]. Here, d corresponds to the accepted absolute error (d) of 5%. According to this formula, a total of 322 samples were required in this study (107 ticks from each governorate (*n* = 3), along with 7 specimens from each herd (*n* = 45)).

The farms visited were small, enclosing an average of twenty heads of cattle, with traditional and poorly maintained dwellings. The cattle analyzed were aged from 6 months to 15 years and mainly belonged to the Friesian Pie Noire and Holstein breeds. Despite the use of an acaricide treatment, almost all animals surveyed were infested with ticks, particularly in the mammary region and the inner surface of the ears. Only unfed and partially engorged ticks were manually collected from different preferred sites of animal bodies (ears, neck, udder and external genitalia) and separately categorized according to the examined cattle. The obtained specimens were morphologically identified using the taxonomic key used by Walker [24], and then classified according to tick species, life stage and gender. Each tick specimen was individually conserved in a tube containing 70% ethanol and stored at −20 °C.

### 2.2. Total DNA Extraction and Tick DNA Amplification

Each identified tick was washed with sterile water, dried and crushed individually using an automated TissueLyser LT system (Qiagen, Hilden, Germany). Genomic DNA extraction was performed from each tick sample using the DNeasy tissue kit (Qiagen, Hilden, Germany). The obtained DNA extracts were stored at −20 °C. The DNA extraction efficiency was validated by PCR amplification targeting the ribosomal RNA subunit (mitochondrial 16S rRNA) gene using the tick-specific primers TQ16S+1F and TQ16S-2R, as described by Black and Piesman [25] (Table 1).

### 2.3. Molecular Detection of Rickettsia Species

Firstly, a nested PCR targeting a fragment (425 bp) of the rickettsial outer membrane protein B (*ompB*) gene tick DNA samples was performed in order to identify all *Rickettsia* species. For further characterization, nested and single PCRs were carried out, respectively, on the outer membrane protein A (*ompA*) and the citrate synthase protein (*gltA*) gene fragments (532 and 381 bp, respectively). The PCR tests were performed in an automated DNA thermal cycler. The thermal cycling profiles were as described by Oteo, Portillo, [27] and Regnery, Olson, [28] respectively.

The PCR reactions were performed in a final volume of 50 µL composed of 0.125 U/µL of Taq DNA polymerase (Biobasic Inc., Markham, Canada), 1× PCR buffer, 0.2 mM of dNTP, 1.5 mM of MgCl_2_, 3 μL of genomic DNA (50–150 ng) in the first PCR and 1 μL in the second PCR (for nested PCR), 0.5 μM of the primers and autoclaved water. An electrophoresis phase in 1.5% agarose gels stained with ethidium bromide was performed to visualize the PCR products under UV transillumination.

### 2.4. Statistical Analysis

The exact confidence intervals (CI) for prevalence rates at the 95% level were estimated. To study the potential influence of abiotic factors (geographic sites and bioclimatic areas) and factors related to the ticks (species and gender) on the molecular prevalence of *Rickettsia* species, a chi-square test or Fisher’s exact test was performed using Epi Info 6.01 (CDC, Atlanta, GA, USA) with a threshold value of 0.05.

### 2.5. DNA Sequencing, Sequence Alignment and Phylogenetic Study

Selected positive PCR products obtained after mitochondrial 16S rRNA, *ompB*, *ompA* and *gltA* PCR tests were selected and purified using the GF-1 Ambi Clean kit (Vivantis, Oceanside, CA, USA) according to the manufacturer’s instructions. The purified DNA amplicons were sequenced in both directions, using the same primers as for the single mitochondrial 16S rRNA and *gltA* PCRs and the second PCR of each nested PCR amplification targeting *ompA* and *ompB* genes. The Big Dye Terminator cycle sequencing ready reaction kit (Applied Biosystems, Foster City, CA, USA) and an ABI3730XL automated DNA sequencer (Macrogen Europe, Amsterdam, The Netherlands) were employed.

The chromatograms were evaluated with Chromas Lite v 2.01 (http://www.technelysium.com.au/chromas_lite.html (accessed on 3 September 2022)). The raw sequences were determined on both forward and reverse strands in order to achieve maximal data accuracy. The complementary strands of each sequenced product were manually assembled using the DNAMAN software program (Version 5.2.2; Lynnon Biosoft, Que., Canada). The overlapping parts were selected after the automatic removal of primer region sequences. The nucleotide sequences of the mitochondrial 16S rRNA as well as of the three genes *ompB*, *ompA* and *gltA* of *Rickettsia* spp. were used to calculate the genotype diversity (Gd), the nucleotide diversity (Pi) and the average number of nucleotide differences (k), using DnaSP version 5.10 software (http://www.ub.edu/dnasp/ (accessed on 3 September 2022)).

Sequence similarities were calculated using the CLUSTAL W method [29] after multiple sequence alignments. A BLAST analysis was performed to assess the level of similarity with previously reported sequences (http://blast.ncbi.nlm.nih.gov/ (accessed on 25 September 2022)). By using the DNAMAN software program, genetic distances among the operational taxonomic units were computed using the maximum composite likelihood method [30] and were used to construct neighbor-joining trees [31]. Using a bootstrapping process with 1000 iterations, the statistical support for the internal branches of the trees was evaluated [32].

## 3. Results

### 3.1. Morphological and Molecular Identification of Ticks and Phylogenetic Analysis

#### 3.1.1. Efficiency of DNA Isolation and Distribution of Collected Ticks

A total of 338 adult ticks (70 females and 268 males) were collected from cattle situated in the Bizerte, Manouba and Ariana governorates, comprising higher semi-arid area (31.9%) and subhumid (22.8%) areas (Table 2). The tick DNA extracts were tested using a single PCR based on mitochondrial 16S rRNA and validated in all samples (100%). The morphological diagnosis using the diagnostic key used by Walker et al. [24] and molecular identification involving sequencing and a BLAST analysis of a partial sequence of the mitochondrial 16S rRNA gene of 83 ticks showed that the 338 collected ticks belong to the two genera *Hyalomma* (*n* = 227) and *Rhipicephalus* (*n* = 111), particularly to the species *Hy. excavatum (n =* 129)*, Rh. sanguineus* sensu lato *(n =* 111), *Hy. marginatum (n =* 84), *Hy. scupense (n =* 12) and *Hy. rufipes (n =* 2) (Table 2).

#### 3.1.2. Genotyping and Phylogenetic Analysis of Selected Tick Specimens

In order to confirm the results of the morphological identification of the analyzed ticks and to genetically characterize the isolates of each revealed species, the sequencing of 320 bp of the mitochondrial 16S rRNA gene was carried out on 83 randomly selected positive (*n* = 56) and negative (*n* = 27) ticks for the *Rickettsia* genus. Four species of the *Hyalomma* genus, namely *Hy. marginatum*, *Hy. excavatum*, *Hy. scupense* and *Hy. rufipes,* and one species of the *Rhipicephalus,* genus namely *Rh. sanguineus* s.l., were identified from the BLAST analysis (Table 3, Tables 5 and 6 and Tables S1–S4). Based on this partial sequence, we accurately selected genotypes that differed from each other by at least one mutation at the nucleotide sequence belonging to the mitochondrial 16S rRNA gene.

**Figure 2:**
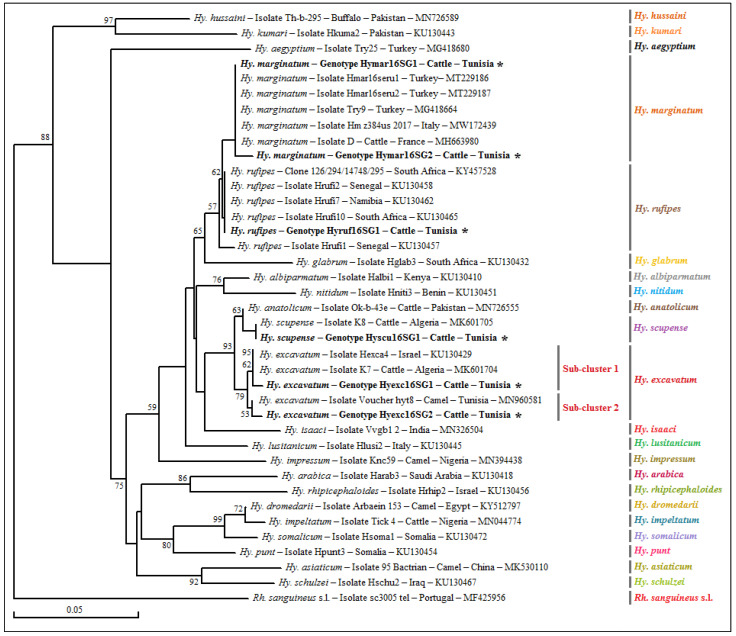
Phylogenetic tree representing partial sequences (320 bp) of the mitochondrial 16S rRNA gene isolated from analyzed tick specimens belonging to *Hy. marginatum*, *Hy. excavatum* and *Hy. rufipes* with those of the *Hyalomma* species published in GenBank using the neighbor-joining method. Legend: Branche-related numbers represent the bootstrap percentages over 1000 iterations supporting the nodes (only percentages greater than 50% are shown). The host, genotype, strain, isolate or clone, country of origin and GenBank accession number are indicated. The sequences of *Rickettsia* spp. newly obtained in this study are represented in bold and marked with an asterisk. A partial sequence of the mitochondrial 16S rRNA gene isolated from a *Rh. sanguineus* s.l. tick was added as an out-group sequence. Note: Our GenBank accession numbers related to each genotype present in the tree are shown in Table 3 and Table 5.

The genetic diversity analysis performed using DnaSP version 5.10.01 software on a 272 bp sequence of the mitochondrial 16S rRNA gene identified two different genotypes for *Hy. marginatum* named Hymar16SG1 and Hymar16SG2, isolated from 24 specimens with genotype diversity (Gd) equal to 0.159. The percentage of GC was 47.8%. The nucleotide diversity (Pi) and the average number of nucleotide differences (k) were estimated, respectively, at 0.00059 and 0.159 by noting the presence of a single mutational position between the two revealed genotypes, sharing 99.63% similarity in terms of nucleotide sequences (Table 4). The first genotype (Hymar16SG1) was found to be different from those published in GenBank and is, therefore, considered a new genetic variant (Table 3 and Table S1). The second genotype (Hymar16SG2) was found to be identical to isolate D of the *Hy. marginatum* tick specimen infesting cattle in France (GenBank accession number MH663980) (Figure 2). The alignment of partial sequences of the mitochondrial 16S rRNA gene of *Hy. excavatum* revealed two different genotypes, named Hyexc16SG1 and Hyexc16SG2, isolated from 31 tick specimens, with genotype diversity (Gd) equal to 0.452. The percentage of GC was 49.3%. The nucleotide diversity (Pi) and average number of nucleotide differences (k) were estimated, respectively, at 0.00502 and 1.355 by noting the presence of three mutational positions between the two revealed genotypes, sharing 98.89% similarity in terms of nucleotides (Table 4). The two genotypes (Hyexc16SG1 and Hyexc16SG2) were found to be different from all those published in GenBank and are, therefore, considered novel genetic variants (Table 5 and Table S2). The only 16S rRNA sequence revealed from *Hy. rufipes* is represented by the Hyruf16SG1 genotype. This genotype is identical to the South African isolate Hrufi10 (GenBank accession number KU130465) (Table 5).

The phylogenetic analysis based on the alignment of Tunisian genotypes belonging to the three revealed tick species, with different sequences of several *Hyalomma* species obtained from GenBank, generated several clusters (Figure 2). The *Hy. marginatum* cluster is formed by several isolates from different Mediterranean countries such as Italy, France and Turkey (Figure 2). The first genotype (Hymar16SG1) was found to be identical to all these isolates, while the second (Hymar16SG2) is genetically close. Moreover, the Hy. excavatum cluster is formed of two subclusters with a node robustness equal to 79% (Figure 2). The first genotype (Hyexc16SG1) was assigned to the first subcluster, with a *Hy. excavatum* isolate from Algeria (MK601704), and the second genotype (Hyexc16SG2) was clustered with an isolate collected from a Tunisian dromedary (GenBank accession number MN960581) in the second subcluster. The cluster representing the *Hy. rufipes* species is composed of the Hyruf16SG1 genotype revealed in the present study and those isolated from other tick specimens of *Hy. rufipes* from several African countries such as Senegal, Namibia and South Africa (Figure 2).

**Figure 3:**
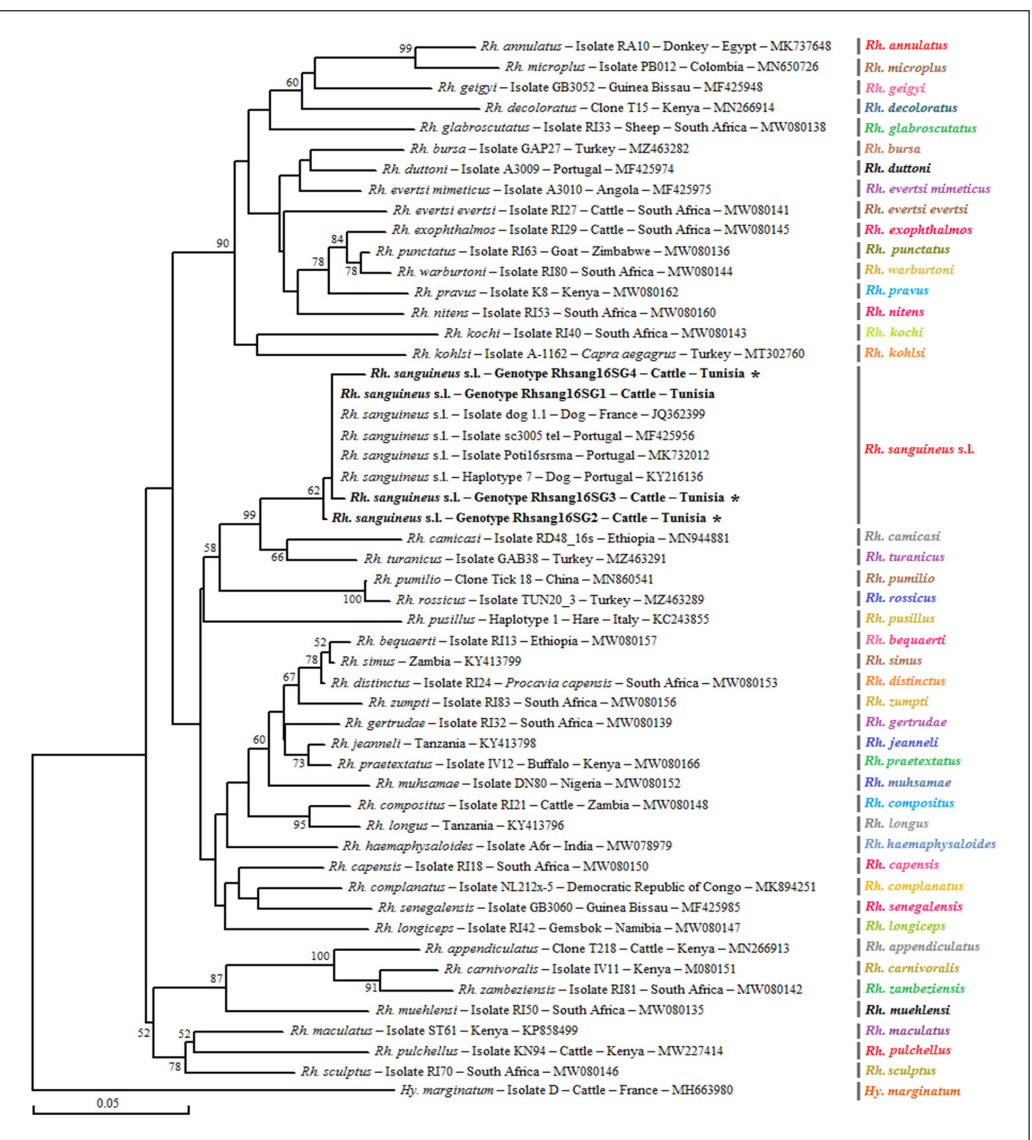
A phylogenetic analysis of partial sequences (320 bp) of the mitochondrial 16S rRNA gene isolated from the revealed tick specimens of the *Rhipicephalus* sanguineus sensu lato complex with those of other *Rhipicephalus* species published in GenBank using the neighbor-joining method. Legend: Branche-related numbers represent the bootstraps rate over 1000 iterations supporting the nodes (only percentages greater than 50% are shown). The host, strain, isolate or clone, country of origin and GenBank accession number are indicated. The sequences of *Rh. sanguineus* s.l. newly obtained in this study are represented in bold and marked with an asterisk. A partial sequence of the mitochondrial 16S rRNA gene isolated from the *Hy. marginatum* tick was added as an out-group sequence. Note: Our GenBank accession numbers related to each genotype are shown in Table 6.

The genetic diversity analysis carried out using the software program DnaSP version 5.10.01 made it possible to identify four different genotypes for *Rh. sanguineus* s.l. named Rhsang16SG1–Rhsang16SG4, isolated from 15 tick specimens, with a diversity of genotypes (Gd) equal to 0.467. The percentage of GC was 48.5%. The nucleotide diversity (Pi) and average number of nucleotide differences (k) were estimated, respectively, at 0.00189 and 0.514 by noting the presence of three mutational positions between the four revealed genotypes, sharing 99.6 to 99.3% nucleotide similarity (Table 4). The first genotype (Rhsang16SG1) was found to be identical to isolate dog 1.1 from a tick of the *Rh. sanguineus* s.l. complex collected from a dog in France (GenBank accession number JQ362399). The remaining three genotypes (Rhsang16SG2–Rhsang16SG4) were found to be different from all of those published in GenBank and are, therefore, considered new genetic variants (Table 4 and Table 6). The phylogenetic analysis based on the alignment of our *Rh. sanguineus* s.l. sequences showed a similarity with those of *Rhipicephalus* spp. published in GenBank. The cluster of *Rh. sanguineus* s.l. was composed of several isolates from southern Mediterranean countries such as Portugal and France. The Rhsang16SG1 genotype was phylogenetically the closest, while the Rhsang16SG2 genotype was the most distant (Figure 3).

### 3.2. Molecular Prevalence of Rickettsia spp.

According to our *ompB* PCR results, the overall infection rate of *Rickettsia* spp. was 26.6% (90/338). The *Rickettsia* infection rates were similar between higher semi-arid (31.9%) and subhumid (22.8%) areas and the low difference between the infection rates was statistically not significant (*p* = 0.063) (Table 2). Additionally, the infection prevalence rates between the governorates were similar, estimated at 22.8%, 32.7% and 28% in cattle ticks located, respectively, in farms from the governorates of Bizerte, Manouba and Ariana, showing a statistically non-significant difference (*p* = 0.157) (Table 2). The four tick species showed distinct infection rates and the difference was statistically significant (*p* = 0.022). Indeed, the highest rate was estimated in *Hy. rufipes* (50%) followed by *Rh. sanguineus* s.l. (34.2%), then finally *Hy. marginatum* and *Hy. excavatum,* with similar rates estimated at 29.8% and 20.1%, respectively (Table 2). Additionally, a statistically non-significant difference was recorded between the prevalence rates in the two sexes (*p* = 0.186), with rates estimated at 32.8% and 25% in female and male ticks, respectively (Table 2).

### 3.3. Rickettsia Species Identification

In order to identify and genetically characterize the revealed *Rickettsia* species, at least one of the three partial sequences of the analyzed genes (*ompB*, *ompA* and *gltA*) was sequenced for the *66* samples positive for *Rickettsia* spp. selected for sequencing (i.e., 22 *Hy. excavatum*, 22 *Rh. sanguineus* s.l., 21 *Hy. marginatum* and one *Hy. rufipes*). Partial sequences (*n* = 104) of the three analysed genes were obtained and deposited in GenBank under the accession numbers OQ123608–OQ123654 for *ompB* partial sequences, OQ123655-OQ123684 for *ompA* partial sequences and OQ123685–OQ123711 for *gltA* partial sequences.

Three *Rickettsia* species were identified in ticks positive for *Rickettsia* spp. selected for the genetic analysis, namely *R. aeschlimannii*, *R. sibirica* subsp. *mongolitimonae* and *R. massiliae* (Table 7). Based on the analysis of the three genes, coinfection by *R. massiliae* and *R. aeschlimannii* was reported in two ticks of *Hy. marginatum* (Hyma72 and Hyma336) and two *Rh. sanguineus* s.l. ticks (Rhsa73 and 273), while a coinfection with *R. massiliae* and *R. sibirica* subsp. *mongolitimonae* was only recorded in one specimen of the *Rh. sanguineus* s.l. complex (Rhsa284) (Table 3, Table 5 and Table 6).

### 3.4. Genotyping and Phylogenetic Analysis

Based on all revealed sequences of the three analysed genes, we precisely selected the genotypes that differed from each other by at least one mutation at the nucleotide sequence level.

#### 3.4.1. *Rickettsia* spp. *ompB* Partial Sequences

The sequencing of *ompB* partial sequence (382 bp) was performed on 47 cattle tick samples belonging to *Hy. marginatum* (*n* = 14), *Hy. excavatum* (*n* = 18), *Hy. rufipes* (*n* = 1) and *Rh. sanguineus* s.l. (*n* = 14). The BLAST analysis showed that 17 *Hy. excavatum*, 13 *Hy. marginatum* and one *Hy. rufipes* tick were infected with *R. aeschlimannii*. In addition, two ticks belonging to *Hy. excavatum* and *Hy. marginatum* species were positive for *R. sibirica* subsp. *mongolitimonae*. Furthermore, the sequencing of fourteen samples of *Rh. sanguineus* s.l. ticks showed that 13 samples were found to be infected with *R. massiliae* and only one tick specimen was positive for *R. sibirica* subsp. *mongolitimonae* (Table 7).

The genetic diversity analysis carried out using DnaSP version 5.10.01 software on a 382 bp of the *ompB* gene made it possible to identify two different genotypes for 13 *R. massiliae* isolates, named Rmas*ompB*G1 and Rmas*ompB*G2, with genotype diversity (Gd) equal to 0.385. The GC rate was 51.3%. The nucleotide diversity (Pi) and average number of nucleotide differences (k) were estimated, respectively, at 0.00503 and 1.923 by noting the presence of 5 mutational positions between the two different revealed genotypes, sharing 98.69% nucleotide similarity (Table 4). These two genotypes were precisely isolated from thirteen specimens of *Rh. sanguineus* s.l. ticks (Table 6). The Rmas*ompB*G1 genotype was found to be identical to the MTU5 strain isolated from a human in France (GenBank accession number CP000683), and the Rmas*ompB*G1 genotype was identical to the Bar29 strain isolated from a *Rh. sanguineus* s.l. tick specimen located in Spain (GenBank accession number AF123710).

The sequence alignment of *R. aeschlimannii* revealed a single genotype named Rae*ompB*G1 isolated from specimens belonging to *Hy. marginatum*, *Hy. excavatum* and *Hy. rufipes*. This genotype was identical to the DoDr354 clone belonging to *R. aeschlimannii* isolated from a *Hyalomma dromedarii* tick specimen infesting a Tunisian camel (GenBank accession number MN094818). The alignment of partial sequences belonging to *R. sibirica* subsp. *mongolitimonae* made it possible to select a single genotype named Rmong*ompB*G1 infecting one *Hy. marginatum*, one *Hy. excavatum* specimen and one *Rh. sanguineus* s.l. tick. This genotype was found to be identical to the pathogenic isolate Urrmtmfee65 of *R. sibirica* subsp. *mongolitimonae* infecting a human from Algeria (GenBank accession number DQ097083).

**Figure 4:**
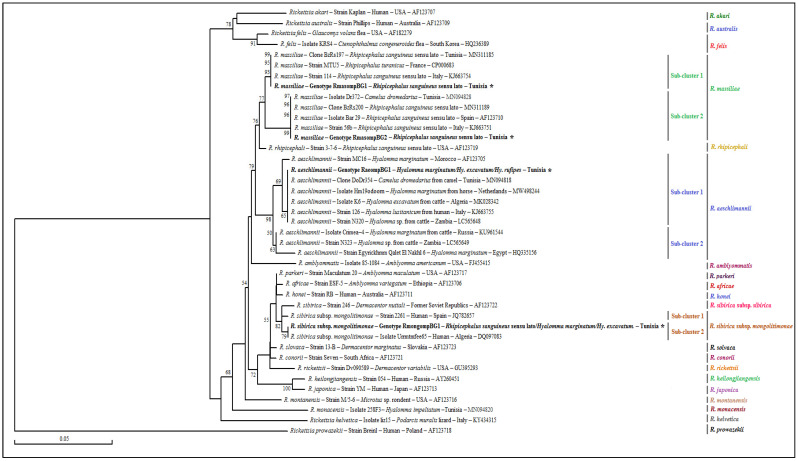
Phylogenetic tree of *Rickettsia* species inferred with partial *ompB* sequences (382 bp) of *Rickettsia* spp. obtained in this study, with selected sequences representative of the *Rickettsia* genus. Legend: Numbers over the branches indicate the percentage of replicated trees in which the associated taxa clustered together in the bootstrap test (1000 replicates, only percentages greater than 50% are represented). The partial *ompB* sequences representative of different *Rickettsia* spp. genotypes obtained in this study are indicated in bold and marked with an asterisk. The host or vector, genotype, strain or isolate name, country of origin and GenBank accession number are indicated. One *R. prowazekii ompB* partial sequence was added as an out-group. Note: Our GenBank accession numbers related to each genotype are shown in Table 3, Table 5 and Table 6.

The phylogenetic analysis based on the alignment of our *ompB* genotypes belonging to the three revealed species, with different partial sequences of several classified *Rickettsia* species obtained from GenBank, generated various clusters (Figure 4). The *R. massiliae* cluster is formed of two subclusters genetically close to the *R. rhipicephali* cluster, with a robustness node equal to 77% (Figure 4). The first genotype (RmasompBG1) was assigned to the first subcluster with those isolated from strain MTU5 infecting a human in France (GenBank accession number CP000683), with clone BzRs197 and strain 114 both isolated from *Rh. sanguineus* s.l. ticks, respectively, in Tunisia and Italy (GenBank accession numbers MN311185 and KJ663754, respectively). The second genotype was classified to the second subcluster, with isolate Dr372 infecting a camel in Tunisia (GenBank accession numbers MN094828) and several isolates and strains infecting *Rh. sanguineus* s.l. ticks from several Mediterranean countries, such as Tunisia, Spain and Italy (Figure 4). The *R. aeschlimannii* cluster is formed by two subclusters with a robustness, node equal to 98% (Figure 4). The only revealed genotype (Rae*ompB*G1) was assigned to the first subcluster, containing several isolates and strains infecting various *Hyalomma* ticks species parasitizing a human in Italy and a cattle and a horse, respectively, in Russia and the Netherlands (Figure 4). Finally, the cluster representing the *R. sibirica* subsp. *Mongolitimonae* subspecies is composed of two subclusters, with a robustness node equal to 82%, the second of which is formed by the Rmong*ompB*G1 genotype, revealed in the present study in one Hy. excavatum tick and the pathogenic isolate (Urrmtmfee65) of *R. sibirica* subsp. *mongolitimonae* infecting an Algerian human (GenBank accession number DQ097083) (Figure 4).

#### 3.4.2. *Rickettsia* spp. *ompA* Partial Sequences

The sequencing of a 490 bp fragment of the *ompA* gene, which corresponds to the 532 bp amplified sequence without the forward and reverse primer sequences, confirmed the presence of *R. massiliae*, *R. aeschlimannii* and *R. sibirica* subsp. *mongolitimonae*. The obtained results affirmed that *Hyalomma* ticks, precisely 7 *Hy. marginatum*, 10 Hy. excavatum and one *Hy. rufipes*, were tested positive for *R. aeschlimannii*. However, only one specimen of *Hy. marginatum* was found positive for *R. massiliae* (Table 7). It was noted that only one tick specimen of the *Hy. marginatum* species was recorded as being positive for *R. sibirica* subsp. *mongolitimonae*. However, for *Rhipicephalus sanguineus* s.l., eight were positive for *R. massiliae* and only one tick specimen of this complex was positive for *R. aeschlimannii* (Table 7).

The alignment of sequences Isolated from *R. massiliae* allowed us to select a single genotype named Rmas*ompA*G1 infecting eight *Rh. sanguineus* s.l. ticks and one *Hy. marginatum* tick specimen. This genotype was found to be identical to the BzRs200 clone of *R. massiliae* isolated from a *Rh. sanguineus* s.l. tick specimen infecting a Tunisian goat (GenBank accession number MN311225).

The genetic diversity analysis performed using the software DnaSP version 5.10.01 on a partial sequence of 490 bp of the *ompA* gene made it possible to identify three different *R. aeschlimannii* genotypes named Rae*ompA*G1, Rae*ompA*G2 and Rae*ompA*G3 isolated from seven *Hy. marginatum* specimens, with ten others belonging to Hy. Excavatum species and one *Rh. sanguineus* s.l. specimen, with the diversity of the genotypes (Gd) estimated at 0.542. The percentage of GC was 53.8%. The nucleotide diversity (Pi) and the average number of nucleotide differences (k) were estimated, respectively, at 0.00123 and 0.605 by noting the presence of two mutational positions between the three different revealed genotypes. Our genotypes shared 99.8–99.6% nucleotide similarity. The Rae*ompA*G1 genotype was found to be identical to the Z98 isolate of *R. aeschlimannii* isolated from *Hy. marginatum* in Italy (GenBank accession number MH532240). The two other genotypes Rae*ompA*G2 and Rae*ompA*G3 were different from all other sequences published in GenBank and were considered as new genetic variants (Table 4, Table 5 and Table 6).

The only sequence belonging to *R. sibirica* subsp. *mongolitimonae* infecting one *Hy. marginatum* tick specimen allowed us to select a single genotype named Rmong*ompA*G1. The BLAST analysis showed that the latter was identical to isolate Ro219 infecting a *Hyalomma* nymph tick collected in Turkey (GenBank accession number MF379301).

**Figure 5:**
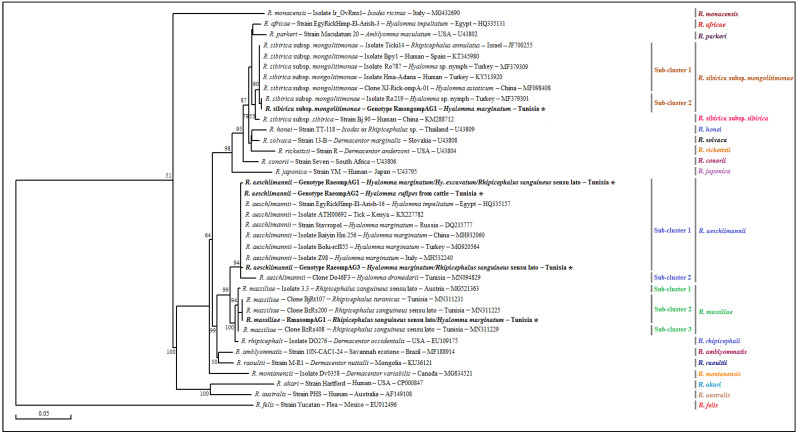
Neighbor-joining tree based on the alignment of partial *ompA* sequences (490 bp) using the neighbor-joining method showing the novel obtained sequences from Tunisian cattle ruminant ticks. Legend: Bootstrap values (1000 replicates) are indicated in each node (only percentages greater than 50% are shown). The genotypes of *Rickettsia* spp. obtained in the present study are indicated in bold and marked with an asterisk. The host or vector, genotype, strain or isolate name, country of origin and GenBank accession number are represented. One *R. felis ompA* partial sequence was added as an out-group. Note: Our GenBank accession numbers related to each genotype are shown in Table 3, Table 5 and Table 6.

The phylogenetic analysis based on the alignment of our Tunisian genotypes with different partial sequences of the *ompA* gene of several *Rickettsia* species obtained from GenBank generated several clusters (Figure 5). The *R. massiliae* cluster comprises three subclusters with a robustness node equal to 94%. The Rmas*ompA*G1 genotype revealed in the present study was assigned to the second subcluster along with those of *R. massiliae* clones BjRt107 and BzRs200 isolated from *Rh. turanicus* and *Rh. sanguineus* s.l. ticks infesting goats in Tunisia (GenBank accession numbers MN311231 and MN311225). The *R. aeschlimannii* cluster is relatively heterogeneous, being composed of three different subclusters, with a robustness node equal to 94%. The three revealed genotypes (Rae*ompA*G1, Rae*ompA*G2 and Rae*ompA*G1) identified in our study were present in the first subcluster, with a multitude of isolates and strains infecting tick specimens mainly of *Hy. marginatum* species from several countries around the world. In the end, the cluster representing the subspecies *R. sibirica* subsp. *mongolitimonae*, which is genetically close to the cluster of *R. sibirica* subsp. *sibirica*, is formed by two subclusters with a robustness node equal to 90%, the second of which is formed by the Rmong*ompA*G1 genotype revealed in this study and the isolate Ro219 infecting a *Hyalomma* sp. tick in Turkey (GenBank accession number MF379301) (Figure 5).

#### 3.4.3. *Rickettsia* spp. *gltA* Partial Sequences

The sequencing of a 341 bp fragment of the *gltA* gene, which corresponds to the 381 bp amplified sequence without the forward and reverse primer sequences, revealed infections with *R. aeschlimannii*, *R. sibirica* subsp. *mongolitimonae* and *R. massiliae* (Table 3, Table 5 and Table 6). The BLAST analysis confirmed the infection of 4 *Hy. excavatum* and 6 *Hy. marginatum* with *R. aeschlimannii*. In addition, one *Hy. marginatum* tick tested positive for *R. massiliae* and two ticks of *Hy. excavatum* and *Hy. marginatum* specimens were found to be infected with *R. sibirica* subsp. *mongolitimonae*. However, 15 *Rh. sanguineus* s.l. ticks were found to be infected with *R. massiliae* (Table 7).

The alignment of sequences belonging to *R. massiliae* revealed a single genotype named Rmas*gltA*G1 infecting 1 and 15 specimens belonging, respectively, to *Hy. marginatum* and *Rh. sanguineus* s.l. This genotype was found to be identical to the *R. massiliae* clone BjRt143 isolated from one *Rh. turanicus* tick infecting a goat from Tunisia (GenBank accession number MW026215). The sequence analysis of *R. aeschlimannii* isolates identified a single genotype named Rae*gltA*G1 infecting six *Hy. marginatum* and four *Hy. excavatum* tick specimens. This genotype was 100% identical to the *R. aeschlimannii* isolate Vc16_16 infecting cattle in France (GenBank accession number MH675648) (Table 3, Table 5 and Table 6). The only sequence belonging to *R. sibirica* subsp. *mongolitimonae* infecting a *Hy. excavatum* tick specimen allowed us to select a single genotype named Rmong*gltA*G1. The BLAST analysis showed that this genotype was 100% identical to the Crimea 2017/2 isolate of *R. sibirica* subsp. *mongolitimonae* infecting one *Hy. marginatum* specimen from Russia (GenBank accession number MT533465).

**Figure 6:**
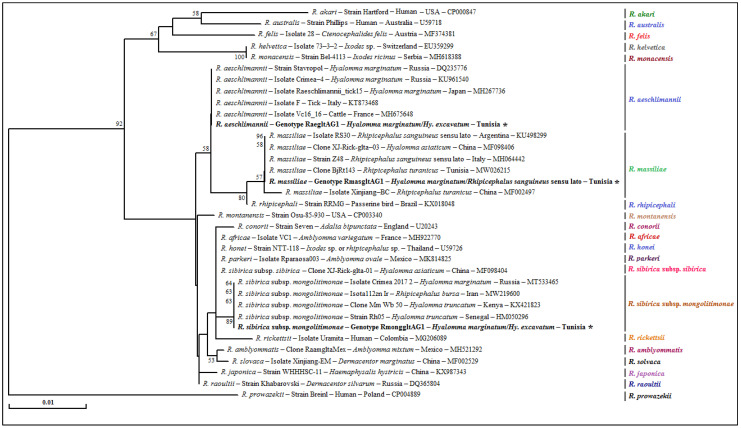
Phylogenetical relationships based on nucleotide multiple alignments of partial *Rickettsia* spp. *gltA* sequences (341 bp). Legend: Numbers over the branches indicate the percentages of replicated trees in which the associated taxa clustered together in the bootstrap test (1000 replicates, only percentages greater than 50% are represented). The only *R. massiliae gltA* genotype revealed in this study from positive samples is represented in bold and marked with an asterisk. The host or vector, genotype, sequence type, strain or isolate name, country of origin and GenBank accession number are indicated. One *R. prowazekii gltA* partial sequence was added as an out-group. Note: Our GenBank accession numbers related to each genotype are shown in Table 3, Table 5 and Table 6.

The phylogenetic tree based on the *gltA* gene revealed that the Rmas*gltA*G1 genotype clustered in the *R. massiliae* cluster with strains infecting *Rh. sanguineus* s.l. ticks from Italy and Argentina, *Hyalomma* asiaticum ticks from China and *Rh. turanicus* tick specimens infesting small ruminants in Tunisia (Figure 6). For the *gltA* gene, the *R. aeschlimannii* cluster is homogeneous and the single genotype (Rae*gltA*G1) revealed in the present study is included with several isolates and strains infecting *Hy. marginatum* specimens from several worldwide countries and with an isolate of *R. aeschlimannii* infecting cattle in France. The cluster representing the subspecies *R. sibirica* subsp. *mongolitimonae* was relatively homogeneous, containing several isolates infecting ticks of *Hy. truncatum* species from African countries and *Hy. marginatum* species located in Russia (Figure 6).

## 4. Discussion

Ticks of the Ixodidae family are, along with mosquitoes, the most relevant vectors of pathogens, with veterinary and medical importance worldwide [33]. The majority of these pathogens appear in tropical countries, which cause the rising of the incidence of tick-borne diseases (TBDs), due to increased interactions between pathogens, hosts and vectors, related directly to global changes [34]. However, epidemiological studies on these diseases are very limited in Tunisia [22].

To date, despite the large cattle population in Tunisia, screening studies for *Rickettsia* bacteria in cattle ticks are very few. Therefore, the present study aimed to detect and characterize ticks of cattle reared in traditional farms located in northern Tunisia and their associated *Rickettsia* species.

In the present study, three hundred and thirty-eight ticks were collected from cattle in northern Tunisia, most of them belonging to the *Hyalomma* genus (227/338), with precisely four species (*Hy. excavatum*, *Hy. marginatum*, *Hy. scupense* and *Hy. rufipes*), while only one *Rhipicephalus* species was identified as *Rh. sanguineus* s.l. (111/338). The overall prevalence of *Rickettsia* spp. was 26.6% (90/338). The tick species *Hy. rufipes* was the most infected (50%), followed by *Rh. sanguineus* s.l. (34.2%) and *Hy. marginatum* (29.8%). The weakest infection rate was recorded in *Hy. excavatum* specimens (20.1%). Indeed, these results concur with those of a recent study conducted on the infection of cattle ticks in Cameroon, reporting a high rate of infection with *Rickettsia* spp. (50%) in *Hy. rufipes* ticks, suggesting that it could be considered as one of the main vectors of *Rickettsia* spp. in this country [35]. Moreover, according to Cicculli et al. [36], *Rickettsia* spp. were detected in *Hy. marginatum* ticks collected from cattle in France (15.5%), suggesting that this tick species has an important role in the transmission of these pathogens. Furthermore, it is interesting to note that other species, such as *Hy. dromedarii* (6%) and *Hy. impeltatum* (8%), are considered potential vectors of *Rickettsia* spp. in camel herds in Tunisia [13]. Additionally, Pesquera et al. [37], as well as Ehlers et al. [38], reported that *Rhipicephalus* ticks, more specifically *Rh. microplus,* are potentially involved in the transmission of *Rickettsia* bacteria to cattle located in Madagascar and the Comoros Islands.

Additionally, the infection rate of *Rickettsia* spp. is less important in *Rh. sanguineus* s.l. compared to those found in other species of the *Hyalomma* genus, in agreement with various reports that have considered that this species of tick is often less infected by *Rickettsia* spp. compared to species belonging to *Hyalomma* and *Amblyomma* genera [35,39,40].

Even if the incidence of TBDs is rising, scarce data on ticks and TBDs in ruminants are available. In Tunisia, previous studies carried out on the detection of the DNA of *Rickettsia* bacteria in ticks collected from small ruminants [22] made it possible to identify the infection rates in *Rh. turanicus* (23.4%) and *Rh. sanguineus* s.l. (9.5%) specimens. This provides evidence that *Rhipicephalus* spp. could be among the main vectors of *Rickettsia* species in northern Tunisia [22]. Our results are also consistent with those reported by Khrouf et al. [21], who suggested the potential incrimination of ticks of the *Rhipicephalus* genus infesting dogs and sheep from central Tunisia in the transmission of *Rickettsia* species.

In the present study, the sequencing of three different DNA fragments of *ompB*, *ompA* and *gltA* genes revealed the presence of three species of *Rickettsia,* namely *R. aeschlimannii*, *R. sibirica* subsp. *mongolitimonae* and *R. massiliae*. The identification of *R. aeschlimannii* in ticks of the *Hyalomma* genus collected from cattle is consistent with the results of previous studies confirming that *Hy. marginatum* and *Hy. excavatum* are the main vectors of this zoonotic agent [41,42,43,44]. *R. sibirica* subsp. *mongolitimonae* was also detected in *Hy. excavatum* and *Rh. sanguineus* s.l. ticks infesting cattle in northern Tunisia. However, this pathogen presents a great topic of interest, since it has been associated with human infections in France [45], South Africa [46], Greece [47] and Spain [48]. Several hypotheses suggest that the tick vectors of this pathogen primarily include species of the *Hyalomma* genus. On the other hand, in addition to *R. aeschlimannii* [49], it seems that other species of *Hyalomma* genus such as *Hy. excavatum* and even species belonging to other genera such as *Rh. sanguineus* s.l. seem to play a role as vectors of this zoonotic bacterium in Mediterranean countries. This diversity of potential tick vectors found in the north of the country could be related to the biotope. Indeed, the latter is characterized by dense vegetation, which offers high protection to ticks and due to its structure prevents the rapid movement of cattle, thereby facilitating their infestation [50]. Moreover, the presence of other animals such as dogs and small ruminants, as well as climate change over the years causing longer periods of drought have led to the abundance and diversification of tick species that infest cattle in these investigated regions [51].

The phylogenetic analysis of our isolates belonging to *R. aeschlimannii* species infecting ticks of the *Hyalomma* genus showed an almost perfect homology with those published in GenBank. Indeed, the analysis of *ompB* and *gltA* partial sequences proved that our isolates are similar to those previously detected in camels in Tunisia [13] and cattle in France [36] and in isolates identified in *Hy. marginatum* ticks from Italy [52]. This finding leads us to suggest that these two animal species as well as their associated tick species, essentially of the *Hyalomma* genus, are probably incriminated in the transmission cycle of *R. aeschlimannii* in the Mediterranean context.

Based on the phylogenetic analysis of *ompB*, *ompA* and *gltA* partial sequences, low genetic diversity was observed among *R. sibirica* subsp. *mongolitimonae* genotypes identified in this study. In particular, the Rmon*ompB*G1, Rmon*ompA*G1 and Rmong*gltA*G1 genotypes of *ompB*, *ompA* and *gltA* genes, respectively, were 100% similar to those isolated from the Urrmtmfee 65, Ro219 and Crimea 2017/2 strains of *R. sibirica* subsp. *mongolitimonae* respectively infecting a human in Algeria, who developed fairly severe symptoms, including an inoculation sore on the leg, fever and lymphangitis extending from the sore to an enlarged and painful lymph node in the groin [53], as well as a tick of *Hyalomma* genus in Turkey [54] and another tick specimen from *Hy. marginatum* in Russia [55]. This finding leads us to suggest that the *R. sibirica* subsp. *mongolitimonae* isolates revealed in the present study could have zoonotic potential with their transmission ensured, in part, by ticks of the *Rh. sanguineus* s.l. complex and those of the *Hyalomma* genus, namely *Hy. excavatum* and *Hy. marginatum*. Despite the fact that the *R. sibirica* subsp. *mongolitimonae* is apparently associated with *Hyalomma* subspecies ticks in North Africa [56], further epidemiological and experimental studies are needed to confirm this hypothesis.

Additionally, *R. massiliae* DNA has been detected in tick specimens belonging to *Rh. sanguineus* s.l, *Hy. marginatum* and *Hy. excavatum*, thereby confirming its presence for the first time in cattle ticks in Tunisia. This SFG *Rickettsia* was identified in many ticks of the *Rhipicephalus* genus, such as *Rh. sanguineus* s.l., *Rh. turanicus* and *Ixodes ricinus* ticks in several European countries, infesting domestic and wild hosts such as dogs, cats, horses, red foxes and asymptomatic humans [57]. The sequence analysis revealed that the *R. massiliae* isolates showed low genetic diversity. In addition, the genotypes identified based on the partial sequences of the *ompB* gene showed perfect similarity to those isolated from the MTU5 strain of *R. massiliae* detected in a human from France [58] and to the Bar29 strain from *Rh. sanguineus* s.l. ticks located in Spain [59]. Furthermore, based on the partial sequence alignment of *ompA* gene, we found that the only revealed genotype (Rmas*ompA*G1) was identical to that of the BzRs200 clone of *R. massiliae* isolated from the *Rh. sanguineus* s.l. tick specimen infecting a Tunisian goat [22]. Based on the *gltA* gene, the only revealed genotype (Rmas*gltA*G1) presented a perfect identity to that previously identified from an isolate of *R. massiliae* infecting one *Rh. turanicus* specimen collected from a goat in Tunisia [22]. Similarly, *R. massiliae* was also identified in *Rh. turanicus* and *Rh. sanguineus* s.l. from Algeria [60], Italy [31], Cyprus [49] and Greece [47]. Based on the analysis of the three genes, the sequence similarity between different isolates and strains of *R. massiliae* infecting humans and several tick species of *Hyalomma* and *Rhipicephalus* genera indicates a possible increased risk of rickettsioses for cattle and even for humans who cohabit in the studied regions.

## 5. Conclusions

In conclusion, the present study provides a molecular survey on *Rickettsia* spp. in cattle ticks from the north of Tunisia. Three *Rickettsia* species (*R. sibirica* subsp. *mongolitimonae*, *R. aeschlimannii* and *R. massiliae*), which are potential or validated human pathogens, were detected and characterized. However, further research studies are necessary to evaluate the pathogenicity of our revealed *Rickettsia* isolates and to confirm the role of each tick species investigated in this study in the transmission of these pathogens to humans and to different animal species in Tunisia.

## Figures and Tables

**Table 1:** Primers used for the identification or genetic characterization of *Rickettsia* species infecting ticks collected from cattle.

Assays (Reference)	Target Genes	Primers	Sequences (5′-3′)	Amplicon Size (bp)
Single PCR ^1^ [25]				
	16S rRNA	TQ16S+1F	CTGCTCAATGATTTTTTAAATTGCTGTGG	324
		TQ16S-2R	ACGCTGTTATCCCTAGAG	
Nested PCR ^2^ [26]				
First PCR	*ompB*	r*ompB*_OF	GTAACCGGAAGTAATCGTTTCGTAA	511
		r*ompB* OR	GCTTTATAACCAGCTAAACCACC	
Second PCR		r*ompB*_SFG_IF	GTTTAATACGTGCTGCTAACCAA	425
		r*ompB* SFG-IR	GGTTTGGCCCATATACCATAAG	
Semi-nested PCR ^3^ [27]				
First PCR	*ompA*	Rr190.70p	ATGGCGAATATTTCTCCAAAA	631
		Rr190.701n	GTTCCGTTAATGGCAGCATCT	
Second PCR		Rr190.70p	ATGGCGAATATTTCTCCAAAA	532
		Rr190.602n	AGTGCAGCATTCGCTCCCCCT	
Single PCR ^3^ [28]				
	*gltA*	RpCS.877p	GGGGGCCTGCTCACGGCGG	381
		RpCS.1258n	ATTGCAAAAAGTACAGTGAACA	

Abbreviations: ^1^ Single PCR based on a mitochondrial 16S rRNA gene allowing the selection of tick samples with DNA extraction efficiency; ^2^ nested PCR based on the *ompB* gene allowing detection or the characterization after the sequencing of *Rickettsia* species; ^3^ single and semi-nested PCR based on *gltA* and *ompA* genes, respectively, allowing the characterization after sequencing of *Rickettsia* species.

**Table 2:** Molecular prevalence results for *Rickettsia* spp. according to tick species, tick gender, bioclimatic area, governorate and district.

Factors	Number (%)	Positive (% ± C.I. ^1^)	*p-*Value (Chi^2^)
Tick species			
*Hyalomma* *excavatum*	129 (38.2)	26 (20.1 ± 0.06)	0.022 ^*^ (11.39)
*Rhipicephalus sanguineus* sensu lato	111 (32.8)	38 (34.2 ± 0.08)	
*Hyalomma* *marginatum*	84 (24.9)	25 (29.8 ± 0.09)	
*Hyalomma* *scupense*	12 (3.6)	0 (0)	
*Hyalomma* *rufipes*	2 (0.6)	1 (50.0 ± 0.69)	
Tick gender			
Male	268 (79.3)	67 (25.0 ± 0.05)	0.186 (1.75)
Female	70 (20.7)	23 (32.8 ± 0.10)	
Bioclimatic area			
Subhumid	197 (58.3)	45 (22.8 ± 0.05)	0.063 (3.45)
Higher semi-arid	141 (41.7)	45 (31.9 ± 0.07)	
Governorate			
Bizerte	197 (58.3)	45 (22.8 ± 0.05)	0.157 (3.70)
Manouba	116 (34.3)	38 (32.7 ± 0.08)	
Ariana	25 (7.4)	7 (28.0 ± 0.17)	
District			
Menzel Bourguiba	113	28 (24.7 ± 0.08)	0.696 (0.152)
Sidi Othman	39	11 (28.2 ± 0.14)	
Mornaguia	35	10 (28.5 ± 0.15)	
Tebourba	31	13 (41.9 ± 0.17)	
Kalaât El Andalous	20	4 (20.0 ± 0.17)	
Djedeida	19	6 (21.5 ± 0.21)	
Bach Hamba	16	0 (0)	
Dhniba	14	4 (28.5 ± 0.24)	
Battan	12	3 (25.0 ± 0.26)	
Mabtouh	10	1 (10 ± 0.19)	
Sejnane	9	1 (11.1 ± 0.2)	
Metline	6	4 (66.66 ± 0.38)	
Bjaoua	5	3 (60.0 ± 0.43)	
Sanhaja	5	2 (40.0 ± 0.43)	
Joumine	3	0 (0)	
Mateur	1	0 (0)	
Total	338	90 (26.6 ± 0.05)	

Abbreviations: ^1^ C.I.: 95% confidence interval; * statistically significant, *p* < 0.05.

**Table 3:** Designation and information on the origins and genotypes of Tunisian isolates of *Rickettsia* spp. isolated from *Hy. marginatum* ticks infesting cattle.

Sample _(District)_	Morp. Id.	BLAST ^1^ (GenBank ^2^, Genotype)	BLAST ^3^ (GenBank ^2^, Genotype)		
			*ompB*	*ompA*	*gltA*
Hyma83 _(Tebourba)_	*Hy.* sp.	100% *Hy. marg* (OQ109189, Hymar16SG1)	100% *R. aesch* (OQ123608, Rae*ompB*G1)	100% *R. aesch* (OQ123655, Rae*ompA*G1)	-
Hyma161 _(Jdaida)_	*Hy. marg*	100% *Hy. marg* (OQ109190, Hymar16SG1)	100% *R. aesch* (OQ123609, Rae*ompB*G1)	-	-
Hyma173 _(Mornaguia)_	*Hy. marg*	100% *Hy. marg * (OQ109191, Hymar16SG1)	100% *R. aesch* (OQ123610, Rae*ompB*G1)	-	100% *R. aesch* (OQ123685, Rae*gltA*G1)
Hyma151 _(Sidi Othmen)_	*Hy. marg*	100% *Hy. marg * (OQ109192, Hymar16SG1)	100% *R. aesch* (OQ123611, Rae*ompB*G1)	-	-
Hyma108 _(Dhniba)_	*Hy. marg*	100% *Hy. marg * (OQ109193, Hymar16SG1)	100% *R. aesch* (OQ123612, Rae*ompB*G1)	-	100% *R. aesch * (OQ123686, Rae*gltA*G1)
Hyma109 _(Dhniba)_	*Hy. marg*	100% *Hy. marg * (OQ109194, Hymar16SG1)	100% *R. aesch* (OQ123613, Rae*ompB*G1)	-	-
Hyma334 _(Metline)_	*Hy. marg*	100% *Hy. marg* (OQ109195, Hymar16SG1)	100% *R. aesch* (OQ123614, Rae*ompB*G1)	-	-
Hyma113 _(Dhniba)_	*Hy. marg*	100% *Hy. marg * (OQ109196, Hymar16SG1)	100% *R. aesch* (OQ123615, Rae*ompB*G1)	100% *R. aesch * (OQ123656, Rae*ompA*G1)	-
Hyma333 _(Metline)_	*Hy. marg*	-	100% *R. aesch* (OQ123616, Rae*ompB*G1)	-	-
Hyma140 _(Sidi Othmen)_	*Hy. marg*	100% *Hy. marg * (OQ109197, Hymar16SG1)	100% *R. aesch* (OQ123617, Rae*ompB*G1)	-	100% *R. aesch* (OQ123687, Rae*gltA*G1)
Hyma96 _(Jdaida)_	*Hy. marg*	100% *Hy. marg* (OQ109198, Hymar16SG1)	100% *R. aesch* (OQ123618, Rae*ompB*G1)	-	100% *R. aesch * (OQ123688, Rae*gltA*G1)
Hyma209 _(Jdaida)_	*Hy. marg*	100% *Hy. marg * (OQ109199, Hymar16SG1)	-	100% *R. aesch* (OQ123657, Rae*ompA*G1)	-
Hyma68 _(Battan)_	*Hy. marg*	99.6% *Hy. marg * (OQ109200, Hymar16SG2)	-	100% *R. aesch * (OQ123658, Rae*ompA*G1)	-
Hyma336 _(Metline)_	*Hy. marg*	100% *Hy. marg * (OQ109201, Hymar16SG1)	-	100% *R. mas * (OQ123676, Rmas*ompA*G1)	-
Hyma25 _(Sejnane)_	*Hy. marg*	-	-	100% *R. aesch * (OQ123659, Rae*ompA*G1)	-
Hyma5 _(K. El Andalous)_	*Hy. marg*	100% *Hy. marg* (OQ109202, Hymar16SG1)	-	100% *R. aesch * (OQ123660, Rae*ompA*G1)	-
Hyma174 _(Mornaguia)_	*Hy. marg*	100% *Hy. marg * (OQ109203, Hymar16SG1)	-	99.8% *R. aesch * (OQ123661, Rae*ompA*G2)	-
Hyma72 _(Tebourba)_	*Hy. marg*	100% *Hy. marg * (OQ109204, Hymar16SG1)	-	-	100% *R. mas * (OQ123696, Rmas*gltA*G1)
Hyma198 _(Tebourba)_	*Hy.* sp.	100% *Hy. marg * (OQ109205, Hymar16SG1)	100% *R. aesch* (OQ123619, Rae*ompB*G1)	-	100% *R. aesch* (OQ123689, Rae*gltA*G1)
Hyma226 _(Tebourba)_	*Hy.* sp.	100% *Hy. marg * (OQ109206, Hymar16SG1)	100% *R. sib* subsp. *mong* (OQ123639, Rmong*ompB*G1)	100% *R. sib* subsp. *mong* (OQ123675, Rmong*ompA*G1)	100% *R. sib* subsp. *mong* (OQ123694, Rmong*gltA*G1)
Hyma156 _(Sidi Othmen)_	*Hy. marg*	*-*	100% *R. aesch* (OQ123620, Rae*ompB*G1)	-	-

Abbreviations: Morp. Id.: morphologically identified tick species; ^1^ BLAST analysis for mitochondrial 16S rRNA partial sequence of ticks; ^2^ GenBank accession number; ^3^ BLAST analysis for *ompB*, *ompA* and *gltA* partial sequences of *Rickettsia* spp.; *Hy. marg*: *Hy. marginatum*; *Hy. exc*: *Hy. excavatum*; *R. aesch: Rickettsia aeschlimannii*; *R. mas: Rickettsia massiliae*; *R. sib* subsp. *mong*: *Rickettsia sibirica* subsp. *mongolitimonae*; -: not sequenced.

**Table 4:** Genetic diversity found within mitochondrial 16S rRNA partial sequences isolated from selected ticks for molecular identification and *ompB*, *ompA* and *gltA* partial sequences isolated from *Rickettsia* spp. infecting ticks.

Gene	Tick or *Rickettsia* Species	Size (pb)	N	VS	GC%	G	Gd	Pi	k
Mito 16S rRNA	*Hy. scupense*	273	12	0	48.7	1	0	0	0
	*Hy. marginatum*	272	24	1	47.8	2	0.159	0.00059	0.159
	*Hy. rufipes*	272	1	0	47.8	1	0	0	0
	*Rh. sanguineus* s.l.	272	15	3	48.5	4	0.467	0.00189	0.514
	*Hy. excavatum*	270	31	3	49.3	2	0.452	0.00502	1.355
*ompB*	*R. massiliae*	382	13	5	51.3	2	0.385	0.00503	1.923
	*R. aeschlimannii*	382	31	0	51.3	1	0	0	0
	*R. sibirica* subsp*. mongolitimonae*	382	3	0	51.3	1	0	0	0
*ompA*	*R. massiliae*	490	9	0	54.0	1	0	0	0
	*R. aeschlimannii*	491	20	2	53.8	3	0.542	0.00123	0.605
	*R. sibirica* subsp*. mongolitimonae*	490	1	0	53.3	1	0	0	0
*gltA*	*R. massiliae*	341	16	0	49.0	1	0	0	0
	*R. aeschlimannii*	341	9	0	49.2	1	0	0	0
	*R. sibirica* subsp*. mongolitimonae*	341	2	0	48.4	1	0	0	0

Abbreviations: Mito 16S rRNA = Mitochondrial 16S rRNA; N = number of analyzed sequences; VS = number of variable sites; GC% = percentage in GC; G = number of genotypes; Gd = genotypic diversity; Pi = nucleotide diversity; k = average number of nucleotide differences.

**Table 5:** Designations and information on the origins and genotypes of Tunisian isolates of *Rickettsia* spp. isolated from *Hy. excavatum* and *Hy. rufipes* ticks infesting cattle.

Sample _(District)_	Morp. Id.	BLAST ^1^ (GenBank ^2^, Genotype)	BLAST ^3^ (GenBank ^2^, Genotype)		
			*ompB*	*ompA*	*gltA*
Hyex143 _(Sidi Othmen)_	*Hy.* sp.	99.2% *Hy. exc * (OQ109213, Hyexc16SG1)	-	99.8% *R. aesch * (OQ123662, Rae*ompA*G2)	-
Hyex206 _(Sanhaja)_	*Hy.* sp.	99.2% *Hy. exc * (OQ109214, Hyexc16SG1)	100% *R. aesch * (OQ123621, Rae*ompB*G1)	99.8% *R. aesch * (OQ123663, Raeomp AG2)	-
Hyex90 _(Battan)_	*Hy. exc*	99.6% *Hy. exc * (OQ109215, Hyexc16SG2)	100% *R. aesch* (OQ123622, Rae*ompB*G1)	-	-
Hyex167 _(Mornaguia)_	*Hy. exc*	99.6% *Hy. exc * (OQ109216, Hyexc16SG2)	100% *R. aesch* (OQ123623, Rae*ompB*G1)	-	-
Hyex141 _(Sidi Othmen)_	*Hy. exc*	99.2% *Hy. exc * (OQ109217, Hyexc16SG1)	100% *R. aesch* (OQ123624, Rae*ompB*G1)	-	-
Hyex115 _(Tebourba)_	*Hy. exc*	99.2% *Hy. exc * (OQ109218, Hyexc16SG1)	100% *R. aesch* (OQ123625, Rae*ompB*G1)	-	-
Hyex16 _(K. El Andalous)_	*Hy. exc*	99.6% *Hy. exc* (OQ109219, Hyexc16SG2)	100% *R. aesch* (OQ123626, Rae*ompB*G1)	-	100% *R. aesch * (OQ123690, Rae*gltA*G1)
Hyex48 _(M. Bourguiba)_	*Hy. exc*	99.6% *Hy. exc * (OQ109220, Hyexc16SG2)	100% *R. aesch* (OQ123627, Rae*ompB*G1)	-	100% *R. aesch * (OQ123691, Rae*gltA*G1)
Hyex78 _(Battan)_	*Hy. exc*	99.6% *Hy. exc * (OQ109221, Hyexc16SG2)	100% *R. aesch* (OQ123628, Rae*ompB*G1)	-	100% *R. aesch * (OQ123692, Rae*gltA*G1)
Hyex171 _(Mornaguia)_	*Hy. exc*	99.6% *Hy. exc * (OQ109222, Hyexc16SG2)	100% *R. aesch* (OQ123629, Rae*ompB*G1)	-	-
Hyex250 _(M. Bourguiba)_	*Hy. exc*	99.2% *Hy. exc * (OQ109223, Hyexc16SG1)	100% *R. aesch* (OQ123630, Rae*ompB*G1)	99.8% *R. aesch * (OQ123664, Rae*ompA*G2)	-
Hyex237 _(Sidi Othmen)_	*Hy. exc*	99.2% *Hy. exc * (OQ109224, Hyexc16SG1)	100% *R. sib* subsp*. mong * (OQ123640, Rmong*ompB*G1)	-	100% *R. sib* subsp*. mong* (OQ123695, Rmong*gltA*G1)
Hyex129 _(Sidi Othmen)_	*Hy. exc*	99.2% *Hy. exc * (OQ109225, Hyexc16SG1)	100% *R. aesch* (OQ123631, Rae*ompB*G1)	-	-
Hyex211 _(Jdaida)_	*Hy. exc*	99.2% *Hy. exc * (OQ109226, Hyexc16SG1)	100% *R. aesch* (OQ123632, Rae*ompB*G1)	99.8% *R. aesch* (OQ123665, Rae*ompA*G2)	-
Hyex195 _(Mornaguia)_	*Hy. exc*	99.2% *Hy. exc * (OQ109227, Hyexc16SG1)	100% *R. aesch* (OQ123633, Rae*ompB*G1)	99.8% *R. aesch * (OQ123666, Rae*ompA*G2)	100% *R. aesch * (OQ123693, Rae*gltA*G1)
Hyex175 _(Mornaguia)_	*Hy. exc*	99.2% *Hy. exc * (OQ109228, Hyexc16SG1)	100% *R. aesch* (OQ123634, Rae*ompB*G1)	99.8% *R. aesch* (OQ123667, Rae*ompA*G2)	-
Hyex188 _(Mornaguia)_	*Hy. exc*	99.2% *Hy. exc * (OQ109229, Hyexc16SG1)	100% *R. aesch* (OQ123635, Rae*ompB*G1)	99.8% *R. aesch * (OQ123668, Rae*ompA*G2)	-
Hyex164 _(Mornaguia)_	*Hy. exc*	99.2% *Hy. exc * (OQ109230, Hyexc16SG1)	-	99.8% *R. aesch * (OQ123669, Rae*ompA*G2)	-
Hyex177 _(Mornaguia)_	*Hy. exc*	99.2% *Hy. exc * (OQ109231, Hyexc16SG1)	100% *R. aesch* (OQ123636, Rae*ompB*G1)	99.8% *R. aesch * (OQ123670, Rae*ompA*G2)	-
Hyex170 _(Mornaguia)_	*Hy. exc*	99.2% *Hy. exc * (OQ109232, Hyexc16SG1)	-	99.8% *R. aesch * (OQ123671, Rae*ompA*G2)	-
Hyex148 _(Sidi Othmen)_	*Hy. exc*	99.2% *Hy. exc * (OQ109233, Hyexc16SG1)	100% *R. aesch* (OQ123637, Rae*ompB*G1)	-	-
Hyru97 _(Jdaida)_	*Hy.* sp.	100% *Hy. ruf* (OQ109244, Hyruf16SG1)	100% *R. aesch* (OQ123638, Rae*ompB*G1)	100% *R. aesch * (OQ123672, Rae*ompA*G3)	-

Abbreviations: Morp. Id.: morphologically identified tick species; ^1^ BLAST analysis for mitochondrial 16S rRNA partial sequence of ticks; ^2^ GenBank accession number; ^3^ BLAST analysis for *ompB*, *ompA* and *gltA* partial sequences of *Rickettsia* spp.; *Hy. marg*: *Hy. marginatum*; *Hy. exc*: *Hy. excavatum*; *Hy. ruf*: *Hy. rufipes*; *R. aesch: Rickettsia aeschlimannii*; *R. mas: Rickettsia massiliae*; *R. sib* subsp. *mong*: *Rickettsia sibirica* subsp. *mongolitimonae*; -: not sequenced.

**Table 6:** Designations and information on the origins and genotypes of Tunisian isolates of *Rickettsia* spp. isolated from *Rhipicephalus sanguineus* sensu lato ticks infesting cattle.

Sample	Morp. Id.	BLAST ^1^ (GenBank ^2^, Genotype)	BLAST ^3^ (GenBank ^2^, Genotype)		
			*ompB*	*ompA*	*gltA*
Rhsa275 _(M. Bourguiba)_	*Rh. sang* s.l.	100% *Rh. sang* s.l. (OQ109257, Rhsang16SG1)	100% *R. mas * (OQ123642, Rmas*ompB*G1)	100% *R. mas* (OQ123677, Rmas*ompA*G1)	100% *R. mas* (OQ123697, Rmas*gltA*G1)
Rhsa282 _(M. Bourguiba)_	*Rh. sang* s.l.	100% *Rh. sang* s.l. (OQ109258, Rhsang16SG1)	100% *R. mas* (OQ123643, Rmas*ompB*G2)	-	100% *R. mas* (OQ123698, Rmas*gltA*G1)
Rhsa77 _(Bjaoua)_	*Rh. sang* s.l.	100% *Rh. sang* s.l. (OQ109259, Rhsang16SG1)	100% *R. mas* (OQ123644, Rmas*ompB*G2)	100% *R. mas* (OQ123678, Rmas*ompA*G1)	100% *R. mas* (OQ123699, Rmas*gltA*G1)
Rhsa1 _(K. El Andalous)_	*Rh. sang* s.l.	99.6% *Rh. sang* s.l. (OQ109260, Rhsang16SG2)	100% *R. mas* (OQ123645, Rmas*ompB*G2)	100% *R. mas* (OQ123679, Rmas*ompA*G1)	100% *R. mas* (OQ123700, Rmas*gltA*G1)
Rhsa73 _(Bjaoua)_	*Rh. sang* s.l.	99.6% *Rh. sang* s.l. (OQ109261, Rhsang16SG3)	100% *R. mas* (OQ123646, Rmas*ompB*G1)	99.8% *R. aesch * (OQ123673, Rae*ompA*G2)	100% *R. mas* (OQ123701, Rmas*gltA*G1)
Rhsa9 _(K. El Andalous)_	*Rh. sang* s.l.	100% *Rh. sang* s.l. (OQ109262, Rhsang16SG1)	100% *R. mas* (OQ123647, Rmas*ompB*G2)	-	100% *R. mas* (OQ123702, Rmas*gltA*G1)
Rhsa284 _(Mornaguia)_	*Rh. sang* s.l.	99.6% *Rh. sang* s.l. (OQ109263, Rhsang16SG4)	100% *R. sib* subsp. *mong* (OQ123641, Rmong*ompB*G1)	100% *R. mas* (OQ123680, Rmas*ompA*G1)	100% *R. mas* (OQ123703, Rmas*gltA*G1)
Rhsa252 _(Sanhaja)_	*Rh. sang* s.l.	100% *Rh. sang* s.l. (OQ109264, Rhsang16SG1)	100% *R. mas* (OQ123648, Rmas*ompB*G1)	-	100% *R. mas* (OQ123704, Rmas*gltA*G1)
Rhsa273 _(M. Bourguiba)_	*Rh. sang* s.l.	99.6% *Rh. sang* s.l. (OQ109265, Rhsang16SG3)	100% *R. mas* (OQ123649, Rmas*ompB*G2)	99.8% *R. aesch* (OQ123674, Raes*ompA*G1)	100% *R. mas* (OQ123705, Rmas*gltA*G1)
Rhsa203 _(Tebourba)_	*Rh. sang* s.l.	100% *Rh. sang* s.l. (OQ109266, Rhsang16SG1)	-	100% *R. mas * (OQ123681, Rmas*ompA*G1)	100% *R. mas* (OQ123706, Rmas*gltA*G1)
Rhsa122 _(Tebourba)_	*Rh. sang* s.l.	-	-	100% *R. mas* (OQ123682, Rmas*ompA*G1)	-
Rhsa121 _(Tebourba)_	*Rh. sang* s.l.	-	-	100% *R. mas* (OQ123683, Rmas*ompA*G1)	100% *R. mas* (OQ123707, Rmas*gltA*G1)
Rhsa254 _(Sanhaja)_	*Rh. sang* s.l.	-	-	100% *R. mas* (OQ123684, Rmas*ompA*G1)	-
Rhsa119 _(Tebourba)_	*Rh. sang* s.l.	-	-	-	100% *R. mas* (OQ123708, Rmas*gltA*G1)
Rhsa120 _(Tebourba)_	*Rh. sang* s.l.	-	-	-	100% *R. mas* (OQ123709, Rmas*gltA*G1)
Rhsa57 _(M. Bourguiba)_	*Rh. sang* s.l.	-	-	-	100% *R. mas* (OQ123710, Rmas*gltA*G1)
Rhsa268 _(Mornaguia)_	*Rh.* sp.	100% *Rh. sang* s. l. (OQ109267, Rhsang16SG1)	100% *R. mas* (OQ123650, Rmas*ompB*G2)	-	-
Rhsa261 _(M. Bourguiba)_	*Rh. sang* s.l.	100% *Rh. sang* s.l. (OQ109268, Rhsang16SG1)	-	-	100% *R. mas* (OQ123711, Rmas*gltA*G1)
Rhsa303 _(M. Bourguiba)_	*Rh. sang* s.l.	*-*	100% *R. mas* (OQ123651, Rmas*ompB*G2)	-	-
Rhsa293 _(M. Bourguiba)_	*Rh. sang* s.l.	*-*	100% *R. mas* (OQ123652, Rmas*ompB*G2)	-	-
Rhsa322 _(M. Bourguiba)_	*Rh. sang* s.l.	*-*	100% *R. mas* (OQ123653, Rmas*ompB*G2)	-	-
Rhsa327 _(M. Bourguiba)_	*Rh. sang* s.l.	*-*	100% *R. mas* (OQ123654, Rmas*ompB*G2)	-	-

Abbreviations: Morp. Id.: morphologically identified tick species; ^1^ BLAST analysis for mitochondrial 16S rRNA partial sequence of ticks; ^2^ GenBank accession number; ^3^ BLAST analysis for *ompB*, *ompA* and *gltA* partial sequences of *Rickettsia* spp.; *Hy. marg*: *Hy. marginatum*; *Hy. exc*: *Hy. excavatum*; *R. aesch: Rickettsia aeschlimannii*; *R. mas: Rickettsia massiliae*; *R. sib* subsp. *mong*: *Rickettsia sibirica* subsp. *mongolitimonae*.

**Table 7:** *Rickettsia* species identified by sequencing partial *ompB*, *ompA* and *gltA* gene sequences infecting cattle ticks.

Tick Species	*ompB* PCR Positive/ Sequenced	*omp*A PCR Positive/Sequenced	*gltA* PCR Positive/Sequenced	*Rickettsia* spp.
*Hyalomma excavatum*	17	10	4	*R. aeschlimannii*
	0	0	0	*R. massiliae*
	1	0	1	*R. sibirica* subsp. *mongolitimonae*
*Hyalomma marginatum*	13	7	5	*R. aeschlimannii*
	0	1	1	*R. massiliae*
	1	1	1	*R. sibirica* subsp. *mongolitimonae*
*Rhipicephalus sanguineus* sensu lato	0	2	0	*R. aeschlimannii*
	13	8	15	*R. massiliae*
	1	0	0	*R. sibirica* subsp. *mongolitimonae*
*Hyalomma rufipes*	1	1	0	*R. aeschlimannii*
	0	0	0	*R. massiliae*
	0	0	0	*R. sibirica* subsp. *mongolitimonae*
Total	47	30	27	*Rickettsia* spp.

## Data Availability

Not applicable.

## Supplementary Materials

The following supporting information can be downloaded at: https://www.mdpi.com/article/10.3390/pathogens12040552/s1, Table S1: Designations and information on the origins and mitochondrial 16S rRNA genotypes of the remaining Tunisian isolates of *Hyalomma marginatum* ticks infesting cattle; Table S2: Designations and information on the origins and mitochondrial 16S rRNA genotypes of the remaining Tunisian isolates of *Hyalomma excavatum* ticks infesting cattle; Table S3: Designations and information on the origins and mitochondrial 16S rRNA genotypes of Tunisian isolates of *Hyalomma scupense* ticks infesting cattle; Table S4: Designations and information on the origins and mitochondrial 16S rRNA genotypes of the remaining Tunisian isolates of *Rhipicephalus sanguineus* sensu lato ticks infesting cattle.
