# Supplementary material for: Diversity and Phylogeny of Cattle Ixodid Ticks and Associated Spotted Fever Group Rickettsia spp. in Tunisia
Source: Pathogens. 2023 Apr 3;12(4):552. doi: 10.3390/pathogens12040552 (PMC10146803; doi:10.3390/pathogens12040552)
Supplement: Supplementary file 1 [file pathogens-12-00552-s001.zip › pathogens-2195011-supplementary.pdf]

pathogens

MDPI

Table S1. Designation, information on the origins, and mitochondrial 16S rRNA genotypes of the remaining Tunisian isolates of *Hyalomma marginatum* ticks infesting cattle.

Sample	Location	*Rickettsia* (+/-)	Morphologically identified tick species	BLAST analysis of mito 16S rRNA (Genotype)
Hyma76	Bjaoua	*Rickettsia* +	*Hyalomma* sp.	100% *Hy. marginatum* (OQ109207, Hymar16SG1)
Hyma2	K. El Andalous	*Rickettsia* -	*Hyalomma* sp.	100% *Hy. marginatum* (OQ109208, Hymar16SG1)
Hyma4	K. El Andalous	*Rickettsia* -	*Hyalomma* sp.	100% *Hy. marginatum* (OQ109209, Hymar16SG1)
Hyma130	Sidi Othmen	*Rickettsia* -	*Hyalomma* sp.	100% *Hy. marginatum* (OQ109210, Hymar16SG1)
Hyma246	M. Bourguiba	*Rickettsia* -	*Hyalomma* sp.	100% *Hy. marginatum* (OQ109211, Hymar16SG1)
Hyma139	Sidi Othmen	*Rickettsia* -	*Hy. marginatum*	99.6% *Hy. marginatum* (OQ109212, Hymar16SG2)

Abbreviations: *Rickettsia* (+/-): Positive or negative to *Rickettsia* spp. *ompB* PCR; K. El Andalous: Kalâat El Andalous; M. Bourguiba : Menzel Bourguiba; mito 16S rRNA: mitochondrial 16S rRNA.

Table S2. Designation, information on the origins, and mitochondrial 16S rRNA genotypes of the remaining Tunisian isolates of *Hyalomma excavatum* ticks infesting cattle.

Sample	Location	*Rickettsia* (+/-)	Morphologically identified tick species	BLAST analysis of mito 16S rRNA (Genotype)
Hyex15	K. El Andalous	*Rickettsia* -	*Hy. excavatum*	99.6% *Hy. excavatum* (OQ109234, Hyexc16SG2)
Hyex93	Jdaida	*Rickettsia* -	*Hy. excavatum*	99.2% *Hy. excavatum* (OQ109235, Hyexc16SG1)
Hyex29	Sejnane	*Rickettsia* -	*Hy. excavatum*	99.6% *Hy. excavatum* (OQ109236, Hyexc16SG2)
Hyex30	Joumine	*Rickettsia* -	*Hy. excavatum*	99.6% *Hy. excavatum* (OQ109237, Hyexc16SG2)
Hyex159	Jdaida	*Rickettsia* -	*Hy. excavatum*	99.2% *Hy. excavatum* (OQ109238, Hyexc16SG1)
Hyex233	Jdaida	*Rickettsia* -	*Hy. excavatum*	99.2% *Hy. excavatum* (OQ109239, Hyexc16SG1)
Hyex138	Sidi Othmen	*Rickettsia* -	*Hy. excavatum*	99.2% *Hy. excavatum* (OQ109240, Hyexc16SG1)
Hyex66	M. Bourguiba	*Rickettsia* -	*Hyalomma* sp.	99.2% *Hy. excavatum* (OQ109241, Hyexc16SG1)
Hyex65	M. Bourguiba	*Rickettsia* -	*Hy. excavatum*	99.6% *Hy. excavatum* (OQ109242, Hyexc16SG2)
Hyex150	Sidi Othmen	*Rickettsia* -	*Hy. excavatum*	99.2% *Hy. excavatum* (OQ109243, Hyexc16SG1)

Abbreviations: *Rickettsia* (+/-): Positive or negative to *Rickettsia* spp. *ompB* PCR; K. El Andalous: Kalâat El Andalous; M. Bourguiba: Menzel Bourguiba; mito 16S rRNA: mitochondrial 16S rRNA.

Table S3. Designation, information on the origins, and mitochondrial 16S rRNA genotypes of Tunisian isolates of *Hyalomma scupense* ticks infesting cattle.

Sample	Location	*Rickettsia* (+/-)	Morphologically identified tick species	BLAST analysis of mito 16S rRNA (Genotype)
Hysc136	Sidi Othmen	*Rickettsia* -	*Hyalomma* sp.	100% *Hy. scupense* (OQ109245, Hyscu16SG1)
Hysc40	Mabtouh	*Rickettsia* -	*Hy. scupense*	100% *Hy. scupense* (OQ109246, Hyscu16SG1)
Hysc38	Mabtouh	*Rickettsia* +	*Hyalomma* sp.	100% *Hy. scupense* (OQ109247, Hyscu16SG1)
Hysc105	Dhniba	*Rickettsia* -	*Hy. scupense*	100% *Hy. scupense* (OQ109248, Hyscu16SG1)
Hysc22	Sejnane	*Rickettsia* -	*Hyalomma* sp.	100% *Hy. scupense* (OQ109249, Hyscu16SG1)

Hysc33	Mabtouh	*Rickettsia* -	*Hy. scupense*	100% *Hy. scupense* (OQ109250, Hyscu16SG1)
Hysc37	Mabtouh	*Rickettsia* -	*Hyalomma* sp.	100% *Hy. scupense* (OQ109251, Hyscu16SG1)
Hysc71	Tebourba	*Rickettsia* -	*Hy. scupense*	100% *Hy. scupense* (OQ109252, Hyscu16SG1)
Hysc103	Dhniba	*Rickettsia* -	*Hyalomma* sp.	100% *Hy. scupense* (OQ109253, Hyscu16SG1)
Hysc104	Dhniba	*Rickettsia* -	*Hyalomma* sp.	100% *Hy. scupense* (OQ109254, Hyscu16SG1)
Hysc106	Dhniba	*Rickettsia* -	*Hyalomma* sp.	100% *Hy. scupense* (OQ109255, Hyscu16SG1)
Hysc42	Mabtouh	*Rickettsia* -	*Hyalomma* sp.	100% *Hy. scupense* (OQ109256, Hyscu16SG1)

Abbreviations: *Rickettsia* (+/-): Positive or negative to *Rickettsia* spp. *ompB* PCR.

Table S4. Designation, information on the origins, and mitochondrial 16S rRNA genotypes of the remaining Tunisian isolates of *Rhipicephalus sanguineus* sensu lato ticks infesting cattle.

Sample	Location	*Rickettsia* (+/-)	Morphologically identified tick species	BLAST analysis of mito 16S rRNA (Genotype)	
Rhsa292	M. Bourguiba	*Rickettsia* +	*Rh. sang* s.l.	100%	*Rh. sang* s.l. (OQ109269, Rhsang16SG1)
Rhsa277	M. Bourguiba	*Rickettsia* +	*Rh. sang* s.l.	100%	*Rh. sang* s.l. (OQ109270, Rhsang16SG1)
Rhsa272	M. Bourguiba	*Rickettsia* +	*Rh. sang* s.l.	100%	*Rh. sang* s.l. (OQ109271, Rhsang16SG1)

Abbreviations: *Rickettsia* (+/-): Positive or negative to *Rickettsia* spp. *ompB* PCR; *Rh. sang* s.l.: *Rhipicephalus sanguineus* sensu lato; M. Bourguiba: Menzel Bourguiba; mito 16S rRNA: mitochondrial 16S rRNA.
